# Effects of dual therapy with betamethasone and tetracycline in a NC/Nga mouse model of atopic dermatitis

**DOI:** 10.3389/jpps.2023.11182

**Published:** 2023-01-19

**Authors:** Katsuhiko Matsui, Mahoka Kobayashi, Mari Nagano, Mio Matsuoka

**Affiliations:** Department of Clinical Immunology, Meiji Pharmaceutical University, Tokyo, Japan

**Keywords:** atopic dermatitis, dual therapy, betamethasone, tetracycline, mouse model, *Staphylococcus aureus*, Langerhans cell

## Abstract

**Purpose:** Our previous study has demonstrated that tetracycline exerts excellent bactericidal activity against *Staphylococcus aureus* strains isolated from patients with atopic dermatitis (AD) while simultaneously inhibiting the development of T helper (Th) type 2 (Th2) cells. The present study was designed to evaluate the effectiveness of dual therapy with betamethasone and tetracycline for AD.

**Methods:** Betametasone (0.1%) and tetracycline (3%) were topically administered to NC/Nga mice with AD-like skin lesions. Skin severity scores, histological changes to the lesioned skin, and serum IgE levels were assessed as indicators of therapeutic effectiveness.

**Results:** Topical treatment with both drugs reduced the skin severity score more significantly than was the case with betamethasone alone or tetracycline alone. This was associated with a reduction in the degree of epidermal thickening, the density of cellular infiltration into the dermis, the mast cell count in the dermis and the serum IgE concentration. Furthermore, the degree of Th1/Th2 cell development in auricular lymph nodes and the *S. aureus* count on the lesioned skin were synergistically suppressed by simultaneous application of both drugs.

**Conclusion:** The present results show that simultaneous topical application of betamethasone and tetracycline synergistically ameliorates AD-like skin lesions in NC/Nga mice. This suggests that dual therapy with betamethasone and tetracycline for AD lesions colonized by *S. aureus* might be one of the best options for inhibiting the development of both Th1 and Th2 cells and acting on superficially located *S. aureus* **.**

## Introduction

Atopic dermatitis (AD) is a chronic inflammatory skin disease with immunopathologic features that vary depending on the duration of the skin lesions (1). Epidemiological studies have shown that AD significantly influences on the quality of life of patients, and its worldwide prevalence is estimated to be about 10%–30% in children and about 2%–10% in adults (2, 3). AD develops early in childhood, and its onset has been linked to multiple genetic abnormalities, a decrease of skin barrier function, immune response abnormality and various environmental factors (4). If the barrier function of the skin is defective, the immune response to the normal skin microbiome is disturbed, and this plays a major role in the pathogenesis of AD (5). The majority of AD patients show superficial skin colonization by the Gram-positive bacterium *Staphylococcus aureus* and increased expression of T helper (Th) type 2 (Th2) cytokines such as interleukin (IL)-4, IL-5 and IL-13 in their peripheral blood mononuclear cells or skin lesions (6, 7). *S. aureus* can be isolated from 96% to 100% of AD skin lesions, whereas only 0–10% of healthy individuals show skin colonization by this organism (8, 9). Furthermore, the frequency of *S. aureus* colonization in the lesional skin of AD patients is markedly higher than that in non-lesional skin, and the *S. aureus* bacterial cell count is also significantly higher in lesional skin than in non-lesional skin (9). We have previously demonstrated that chronic skin colonization with *S. aureus* may augment the development of Th2 cells in AD patients (10), and that *S. aureus* cell wall components induce an increase in the numbers of eosinophils, mast cells and Th2 cells, which are closely associated with exacerbation of skin inflammation in the lesional skin (11, 12, 13, 14). Therefore, any antibiotic effective for AD patients should ideally benefit not only those with impetiginized AD but also those without clinical signs of superinfection. In our previous study, we found that tetracycline exerted excellent bactericidal activity against *S. aureus* strains isolated from AD skin lesions and simultaneously suppressed Th2 cell development mediated by Langerhans cells (LCs), with effects equivalent to those of betamethasone (15, 16). Therefore, in the present study using a model of AD in NC/Nga mice, we evaluated the effect of dual therapy with betamethasone and tetracycline on AD lesions.

## Methods

### Mice

The mice used in this study were 6-week-old female specific pathogen-free NC/Nga mice obtained from Japan SLC (Hamamatsu, Japan). They were housed in plastic cages with sterilized paper bedding in a clean, air-conditioned room at 24°C and allowed free access to a standard laboratory diet and water. All procedures performed on the mice were in accordance with the guidelines of the Animal Care and Use Committee of Meiji Pharmaceutical University and approved by the committee.

### Reagents

A mite antigen, *Dermatophagoides farinae* extract (Biostir AD), was purchased from Biostir Inc. (Osaka, Japan). Betamethasone and tetracycline were obtained from Tokyo Chemical Industry (Tokyo, Japan). White petrolatum including 5% (w/w) liquid paraffin was employed as the vehicle and used to prepare 0.1% (w/w) betamethasone ointment and 3% (w/w) tetracycline ointment.

### Induction of AD-like skin lesions

**FIGURE 1:**
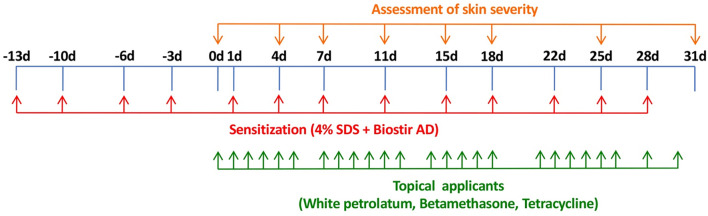
Experimental schedule for induction of AD-like dermatitis in NC/Nga mice and treatment with betamethasone and tetracycline ointment.

The dorsal hair of NC/Nga mice was shaved with a hair clipper and finally removed with a depilatory cream. Skin barrier disruption was then achieved by topical application of 150 μL of 4% sodium dodecyl sulfate to the dorsal skin (120 μL/8 cm^2^ skin) and the bilateral auricle skin (15 μL/1 cm^2^ skin). After 3 h, 100 mg Biostir AD was applied topically to the dorsal skin (80 mg/8 cm^2^ skin) and the bilateral auricle skin (10 mg/1 cm^2^ skin). These procedures were repeated every 3 or 4 days. The design of the experimental schedule is shown in Figure 1.

### Measurement of skin severity score

Dermatitis severity was assessed macroscopically using the scoring system described previously (17). Briefly, one skin lesion (1 cm^2^) on each ear and one skin lesion (8 cm^2^) on the back were scored on the basis of the following criteria. The dermatitis score (minimum 0; maximum 30 [=3 regions × 2 points × 5 symptoms]) was calculated as the sum of the individual scores for the three regions, and graded as 0 (no symptoms), 1 (less than 1/3 of the skin area) or 2 (1/3 and more of the skin area), for each of the following five symptoms: redness/scratch marks, edema/lichenification/thickening, hemorrhage/scabbing, erosion, and desquamation.

### Topical application of betamethasone and tetracycline to NC/Nga mice

After the skin severity score had reached approximately 8–11, corresponding to moderate AD-like skin inflammation, 0.1% betamethasone ointment and 3% tetracycline ointment were applied topically to the dorsal skin and the bilateral auricle skin (50 mg/body [=50 mg/10 cm^2^ skin]) in each group (6 mice per group) once per day, except Sunday and any public holiday. The design of the experimental schedule is summarized in Figure 1.

### Histopathological observations

The dorsal skin was removed 31 days after assessment of skin severity, fixed in 4% paraformaldehyde, embedded in paraffin, and sectioned at a thickness of 5 μm. Tissue sections were then stained with hematoxylin-eosin (HE) and toluidine blue (TB), respectively, and observed microscopically.

### Measurement of serum total IgE levels

Blood samples were collected from the heart 31 days after assessment of skin lesion severity. The total IgE concentration in the serum was measured by enzyme-linked immunosorbent assay (ELISA) using a mouse IgE ELISA kit (Cedarlane, ON, Canada).

### Quantification of Th1 and Th2 cytokine production from T lymphocytes in lymph nodes

Auricular lymph node cells were harvested on the 31st day of skin severity assessment and adjusted to 1 × 10^6^ cells/mL in RPMI 1640 medium with L-glutamine (Sigma-Aldrich, St. Louis, MO, United States) containing 10% fetal bovine serum (Sigma-Aldrich), 25 mM Hepes (Sigma-Aldrich), 100 U/mL penicillin and 100 μg/mL streptomycin (Gibco RBL, Grand Island, NY, United States). The cultures (0.2 mL/well) were incubated in 96-well culture plates (Nunc, Roskilde, Denmark) in the presence of Dynabeads Mouse.

T-Activator CD3/CD28 (Life Technologies, Oslo, Norway) at 37°C in a humidified atmosphere with 5% CO_2_. The culture supernatants were collected after incubation for 48 h, and the interferon (IFN)-γ and IL-4 concentrations were measured using ELISA kits for quantification of murine IFN-γ and IL-4, respectively (R&D Systems, Minneapolis, MN, United States).

### Detection of *S. aureus* on skin

Bacterial isolates were obtained from each skin lesion by applying a “Film Stamp” for 10 s to the affected dorsal skin (8 cm^2^ area [=2 cm × 4 cm]) (9). After an overnight culture on tryptic soy agar plates at 37°C, the colonies grown were characterized by color and diameter, and colony numbers were expressed as colony-forming units (CFUs) per 8 cm^2^ skin area. Microscopic examination of Gram-stained colonies and the PS Latex (Eiken Chemical, Tokyo, Japan) slide agglutination test were also carried out to identify these organisms. *S. aureus* was finally identified from the reaction profile based on the 20 biochemical tests included in the API STPH system (Biomérieux, Marcy-I’Etoile, France).

### Statistical analysis

The data were expressed as means (± standard error of the mean [SEM]), and differences between means were analyzed using one-way ANOVA, followed by Tukey-Kramer multiple comparison test. Differences at *p* < 0.05 were considered to be statistically significant.

## Results

### Therapeutic effects of topically applied betamethasone and tetracycline on AD-like skin lesions in NC/Nga mice

**FIGURE 2:**
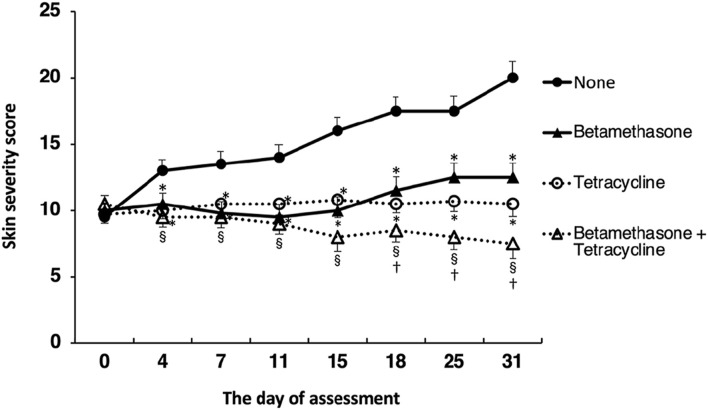
Effects of topically applied betamethasone and tetracycline on skin severity score. The results for each experimental group are expressed as means ± SEM (*n* = 6). *p < 0.01 versus none, ^§^ *p* < 0.01 versus none, ^†^ *p* < 0.05 versus betamethasone, tetracycline.

**FIGURE 3:**
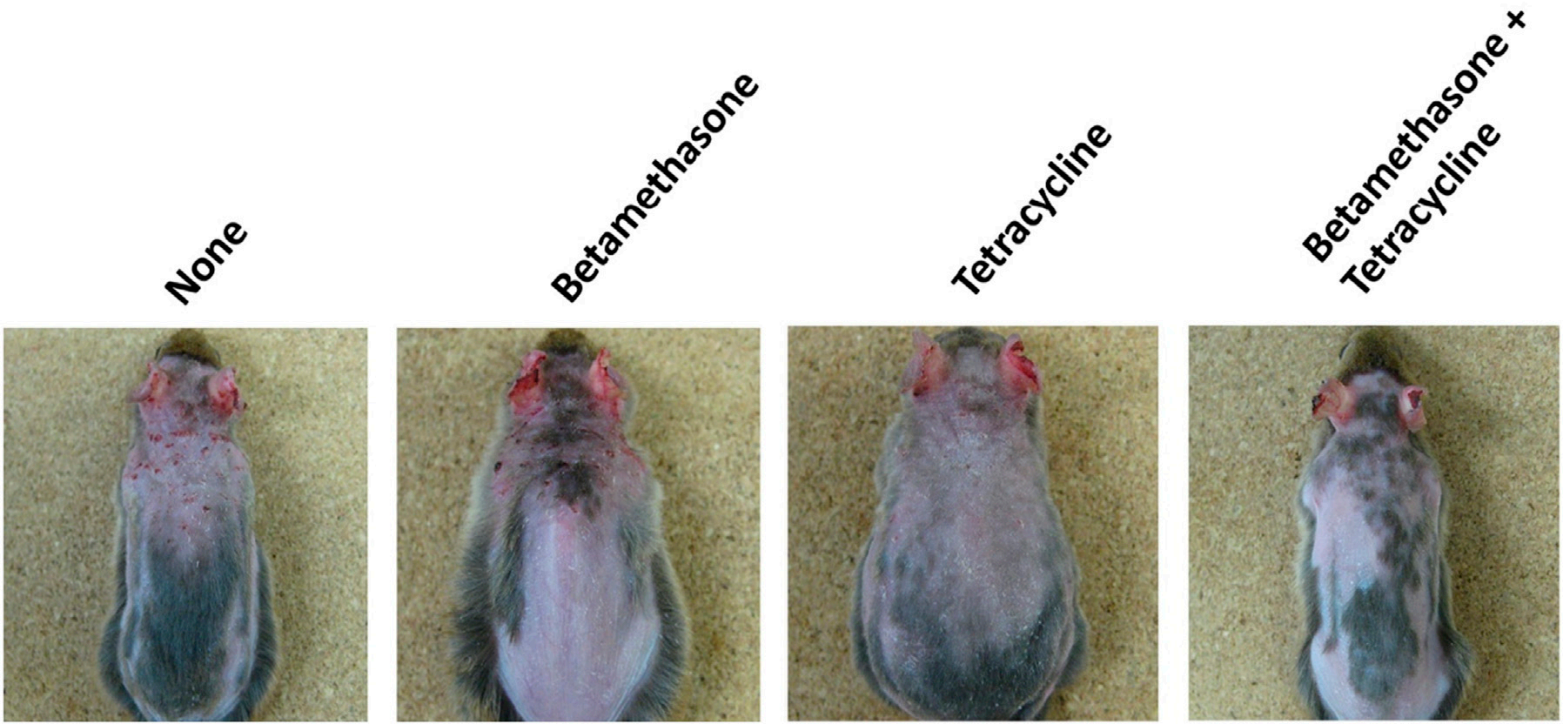
Macroscopic features of AD-like skin lesions in NC/Nga mice. The photograph shows skin lesions on the 31st day of assessment.

Assessment of skin lesion severity in NC/Nga mice sensitized with a mite antigen and medication with ointment was started 13 days after initial sensitization with the mite antigen (Figure 1). Before treatment, the sensitized mice had moderate AD-like skin lesions, and the clinical skin lesion severity in the mite antigen-sensitized control mice (none) increased gradually with each successive assessment (Figure 2). Topical application of vehicle alone did not affect the skin severity score in NC/Nga mice (data not shown), as had also been observed in our previous study (17). However, monotherapy with either betamethasone ointment or tetracycline ointment alone began to suppress the increase in the skin severity score from the fourth day of assessment, although no significant difference between the therapeutic effects of the two drugs was apparent. On the other hand, the therapeutic efficacy of betamethasone ointment and tetracycline ointment in combination became apparent after 18 days of assessment, and its efficacy significantly exceeded that of monotherapy with either ointment alone, persisting throughout the experimental period. All mice in the control group (none) on the 31st day of assessment had AD-like skin lesions comprising redness/scratch marks, edema/lichenification/thickening, hemorrhage/scabbing, erosion and desquamation, and the difference in efficacy between the monotherapy and the combination therapy was macroscopically evident (Figure 3).

### Effects of topical application of betamethasone and tetracycline on histopathological changes in dorsal skin

**FIGURE 4:**
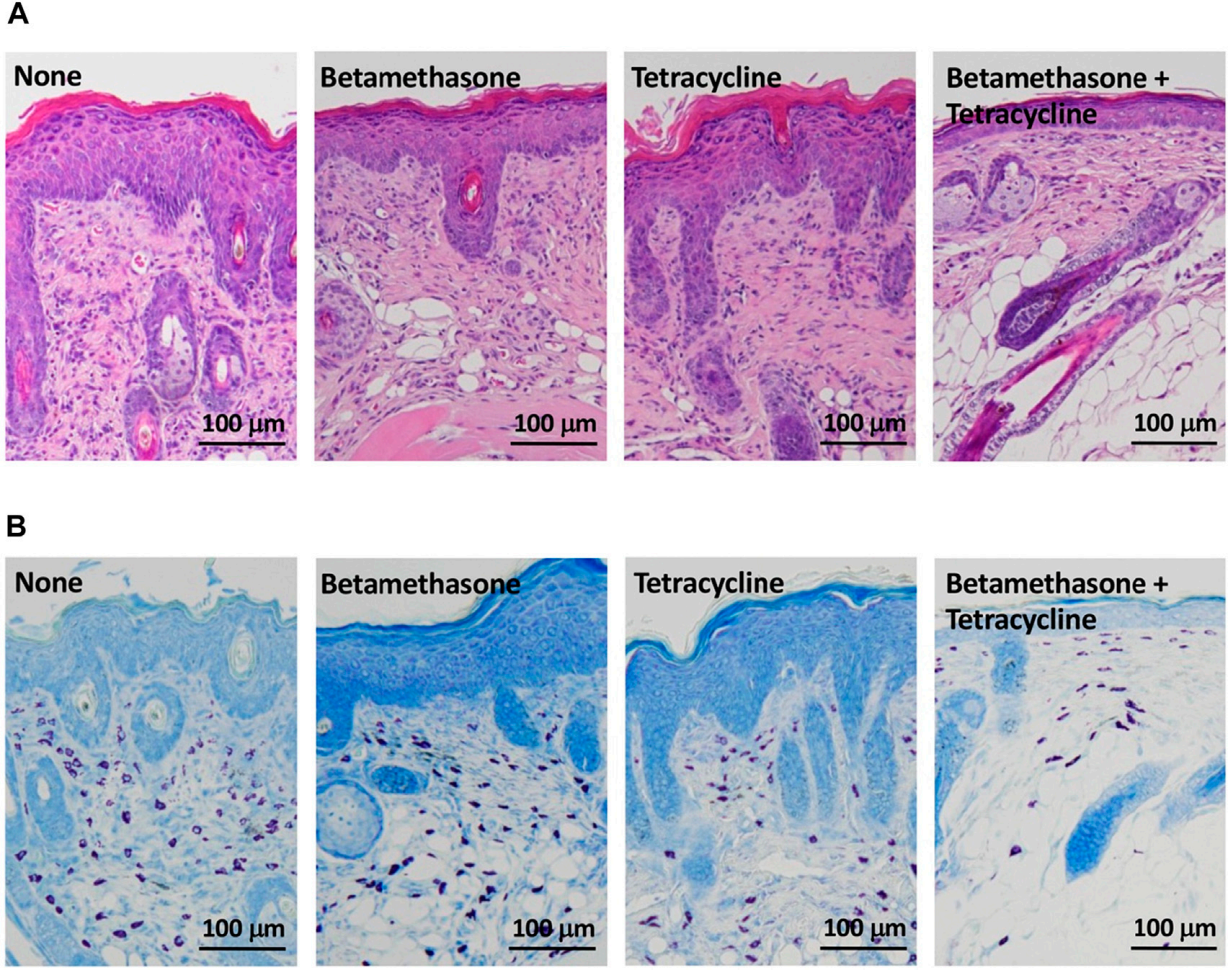
Histopathological analysis of AD-like skin lesions in NC/Nga mice. Skin sections were stained with hematoxylin-eosin **(A)** and toluidine blue **(B)** and observed at ×100. The photograph shows sections of skin lesions on the 31st day of assessment.

As shown in Figure 4, control mice (none) on the 31st day of skin lesion severity assessment showed epidermal hyperplasia and dense infiltration of inflammatory cells such as mast cells, eosinophils and lymphocytes, similar to the skin lesions of AD patients (Figure 4A). Topical application of betamethasone ointment alone or tetracycline ointment alone each had a slightly inhibitory effect on dermal infiltration of inflammatory cells. However, the level of this inhibition elicited by combined topical application of betamethasone ointment and tetracycline ointment was superior to that elicited by betamethasone alone or tetracycline alone. Specifically, the number of mast cells was markedly reduced by betamethasone and tetracycline in combination (Figure 4B). Similarly, the two ointments applied together also reduced the degree of epidermal hyperplasia.

### Effects of topically applied betamethasone and tetracycline on the serum total IgE level

**FIGURE 5:**
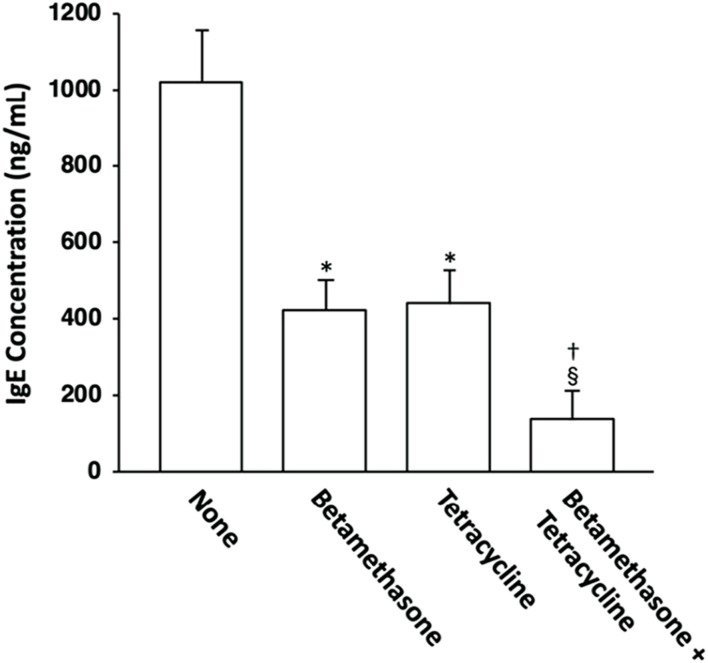
Effects of topically applied betamethasone and tetracycline on serum IgE concentration in NC/Nga mice with AD-like skin lesions. The serum total IgE levels on the 31st day of assessment were measured by ELISA. The results for each experimental group are expressed as means ± SEM (*n* = 6). *p < 0.01 versus none, ^§^ *p* < 0.01 versus none, ^†^ *p* < 0.01 versus betamethasone, tetracycline.

The possible correlation between skin lesion severity and the serum IgE level was then investigated. The serum IgE level was elevated in mite antigen-sensitized NC/Nga mice, and topical application of vehicle alone did not affect it (data not shown). However, the serum total IgE level in control (untreated) mice on the 31st day of skin lesion severity assessment was significantly reduced to almost the same level by topical application of betamethasone alone or tetracycline alone, and combined application of betamethasone and tetracycline further reduced the IgE level relative to each agent used alone (Figure 5).

### Effects of topical application of betamethasone and tetracycline on Th1 and Th2 cell development

**FIGURE 6:**
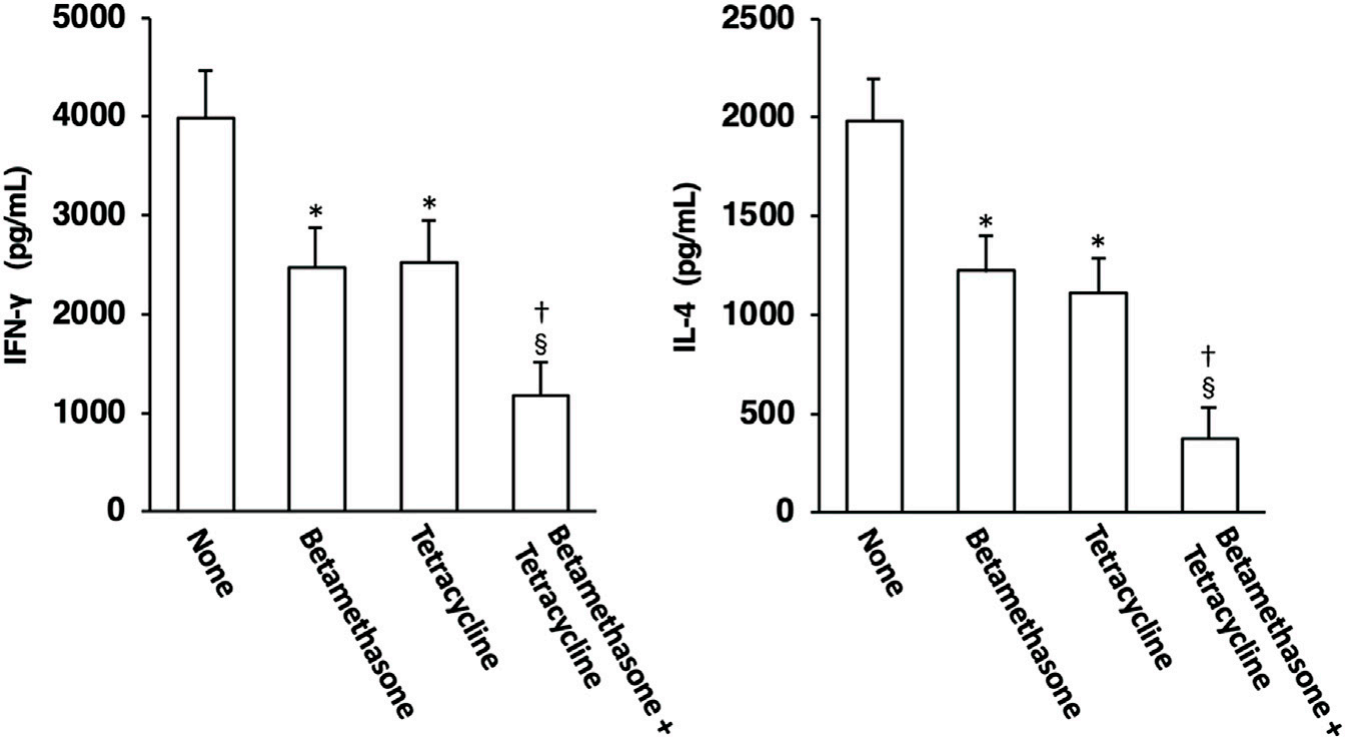
Effects of topically applied betamethasone and tetracycline on Th1 and Th2 cell development in auricular lymph nodes of NC/Nga mice with AD-like skin lesions. Auricular lymph node cells on the 31st day of skin severity assessment were stimulated through their surface CD3/CD28 molecules, and the concentrations of IFN-γ and IL-4 in the culture supernatants were determined by ELISA. Each culture was prepared in triplicate, and the mean value was obtained as a representative result for one experiment. The results are expressed as means ± SEM (*n* = 6). *p < 0.01 versus none, ^§^ *p* < 0.01 versus none, ^†^ *p* < 0.05 versus betamethasone, tetracycline.

The possible correlation between skin lesion severity and the level of Th1 and/or Th2 cell development was then examined. On the 31st day of skin severity assessment, ELISA for IFN-γ and IL-4 was carried out using culture supernatants of lymph node cells that had been stimulated *via* their cell surface CD3/CD28 molecules for 48 h in order to examine Th1 and Th2 cell development in auricular lymph nodes. As shown in Figure 6, elevated production of the Th1 cytokine IFN-γ and the Th2 cytokine IL-4, which serve as indices of Th1 and Th2 cell development, respectively, in lymph node cells from control (untreated) mice was significantly suppressed to almost the same level by topical treatment with betamethasone alone or tetracycline alone. However, topical application of vehicle alone did not affect the increased levels of Th1 and Th2 cell development observed in NC/Nga mice (data not shown), as had been seen in our previous study (17). In addition, the degree of inhibition of Th1 and Th2 cell development by combined application of betamethasone and tetracycline markedly exceeded that resulting from monotherapy with each drug.

### Effect of topical application of betamethasone and tetracycline on skin colonization by *S. aureus*

**FIGURE 7:**
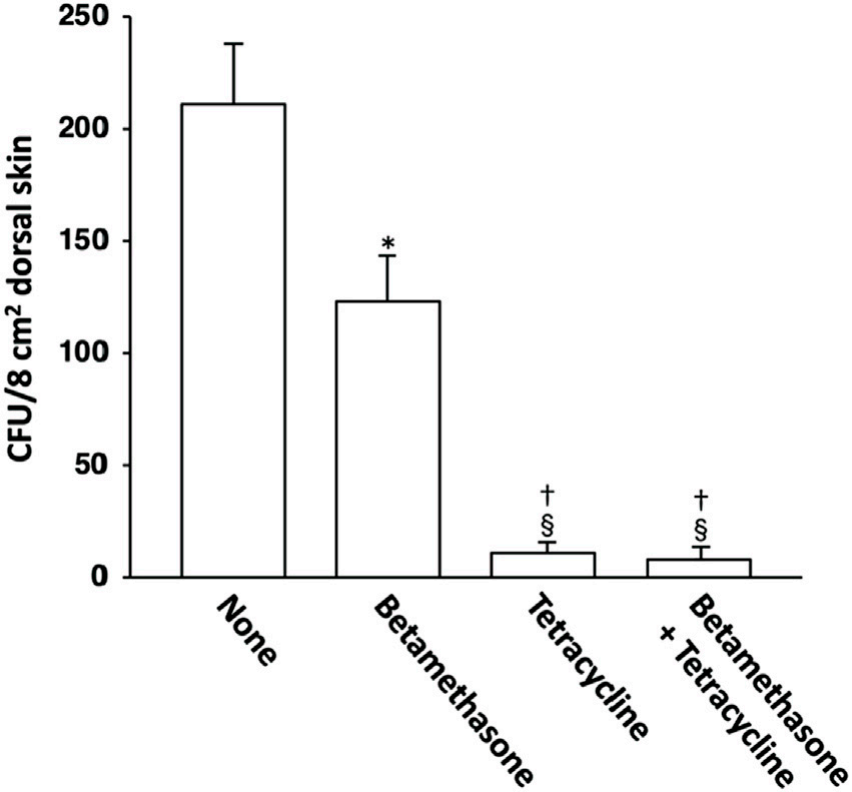
Effects of topically applied betamethasone and tetracycline on bacterial cell count of *S. aureus* on AD-like skin lesions in NC/Nga mice. The results for each experimental group are expressed as means ± SEM (*n* = 6). *p < 0.01 versus none, ^§^ *p* < 0.01 versus none, ^†^ *p* < 0.01 versus betamethasone.

The possible correlation between skin lesion severity and the degree of *S. aureus* skin colonization was then examined. The *S. aureus* CFU count per 8 cm^2^ area of dorsal skin in NC/Nga mice on the 31st day of skin severity assessment is shown in Figure 7. The lesioned skin of control (untreated) mice was heavily colonized by *S. aureus*. In comparison, the normal skin of NC/Nga mice was not colonized with this pathogen (data not shown). Topical application of betamethasone ointment to the lesioned skin decreased the *S. aureus* CFU values slightly.

However, when tetracycline was used alone or in combination with betamethasone, skin colonization by *S. aureus* was suppressed very strongly in comparison to treatment with betamethasone alone. We had already confirmed in our previous study that topical application of vehicle alone did not affect the degree of *S. aureus* colonization in NC/Nga mice (18).

## Discussion

Th1/Th2 immune balance is closely connected with various immunological diseases, including allergy. It is well known that Th2 immunity is responsible for allergic immune responses and the subsequent pathogenesis of allergic diseases (19, 20, 21). AD is one such allergy-related disease, characterized by a marked increase in the count of Th2 cells in both peripheral blood and acute skin lesions (6). On this basis, it has been proposed that the Th2 immune response plays a key pathogenetic role in AD, and this is supported by the presence of blood eosinophilia and enhanced serum IgE levels in most AD patients (22, 23). Our previous study also suggested that the Th1 immune response also plays an instrumental role in the induction of AD-related chronic inflammation (15). Therefore, we have recently been focusing on an immunoregulatory approach for prevention of Th1 and Th2 cell development in AD patients.

Here we demonstrated that a combination of betamethasone and tetracycline ointment markedly suppressed the exacerbation of AD-like skin lesions in a NC/Nga mouse model, with a therapeutic efficacy superior to that of betamethasone alone or tetracycline alone. In comparison to the untreated group, skin lesion severity assessed macroscopically in terms of redness/scratch marks, edema/lichenification/thickening, hemorrhage/scabbing, erosion, and desquamation was significantly decreased by simultaneous application of 0.1% betamethasone ointment and 3% tetracycline ointment. These findings were also supported by histopathologic analysis. Combined topical application of betamethasone and tetracycline inhibited epidermal hyperplasia and dense infiltration of inflammatory cells such as mast cells, eosinophils, and lymphocytes more effectively than betamethasone alone or tetracycline alone. We had shown previously that LCs treated with betamethasone inhibited both Th1 cell and Th2 cell development in lymph nodes, and that LCs treated with tetracycline inhibited only Th2 cell development (15, 16). However, the present study showed that monotherapy with tetracycline also inhibited the development of Th1 cells in addition to Th2 cells, in a manner similar to betamethasone (16). This may have been due to the difference in the concentration of tetracycline between the two studies: in the previous study, 0.1% tetracycline had been used (15), whereas in the present study 3% tetracycline was used to ensure a better therapeutic effect. This suggested that the high concentration of tetracycline controlled not only Th2 cell but also Th1 cell development. The concentrations of the drugs in the ointments (0.1% betamethasone and 3% tetracycline) were equivalent to those used in clinical practice. Therefore, this type of dual therapy with betamethasone and tetracycline would be applicable to actual AD patients. Since it was considered that combined topical application of betamethasone and tetracycline to skin lesions of NC/Nga mice would target LCs in the epidermis, the affected LCs would then move to lymph nodes, where Th1 cell development and subsequent IFN-γ production, as well as Th2 cell development and subsequent IL-4 production, would be synergistically downregulated. This was appropriately reflected in the levels of Th1/Th2 cytokines in auricular lymph nodes on day 31 of skin severity assessment. It is known that the Th2 cytokine response is dominant in the acute phase of AD, and that in the late phase the Th1 cytokine response is also increased, contributing to chronic skin inflammation (6, 22, 24). These facts indicate that combined topical application of betamethasone and tetracycline can synergistically regulate both acute and chronic inflammation, thus contributing to amelioration of AD-like skin lesions in NC/Ng mice.

About 70%–80% of patients with AD show an increased serum level of IgE, which is associated with disease severity (6). An elevated serum IgE level was also observed in mite antigen-treated NC/Nga mice, and topical application of either betamethasone alone or tetracycline alone significantly reduced the serum IgE concentration. IL-4 receptor-mediated signaling in B cells is essential for induction of IgE synthesis (25). Therefore, the reduction in the serum IgE level in mite antigen-treated NC/Nga mice upon topical application of betamethasone or tetracycline could be explained by the degree of IL-4 expression in lymph nodes. Furthermore, topical application of both betamethasone and tetracycline synergistically reduced the serum IgE concentration and IL-4 expression level in NC/Nga mice. The difference of therapeutic efficacy between topical application of either betamethasone alone or tetracycline alone and that achieved by a combination of the two could be explained in terms of the serum IgE concentration, which precisely reflects the degree of Th2 immune response in the living body.

On the other hand, we have shown previously that *S. aureus* isolated from the lesional skin of AD patients is susceptible to tetracycline (15). Since in most AD patients the skin shows superficial *S. aureus* colonization and barrier disruption due to reduction of ceramide or filaggrin (26, 27), bacterial products such as staphylococcal enterotoxins, lipoteichoic acid and peptidoglycan would likely penetrate the skin and induce the production of Th2 cells, and in turn Th1 cells and related chemokines, leading to a Th2 immune response and a subsequent Th1 immune response, thus augmenting skin inflammation (6, 10, 11, 14, 28). Therefore, topical application of tetracycline to the skin lesions of AD patients appears to exert a marked effect involving a bactericidal action against *S. aureus* itself and inhibition of the Th1 and Th2 immune response through inhibition of the LC-mediated allergen-specific Th1 and Th2 cell development (15). Our recent study demonstrated heavy *S. aureus* colonization on the lesional skin of NC/Nga mice and showed that the count of this pathogen on the lesioned skin was correlated with lesion severity (18). Elimination of *S. aureus* colonization is important because otherwise it may trigger skin infection and subsequent impetigo. The results of the present study have shown that tetracycline alone acts against both *S. aureus* and dermatitis. Accordingly, topical administration of tetracycline would control both skin lesion severity and *S. aureus* colonization. Although treatment with betamethasone alone also decreased the *S. aureus* cell count on the lesional skin of NC/Nga mice, this reflected the steroidal effect of betamethasone, and not any intrinsic antibacterial activity. However, we confirmed that *S. aureus* colonization was eliminated more effectively by using an ointment containing both betamethasone with tetracycline than one containing betamethasone alone, which would be largely explainable by the antibacterial effect of tetracycline, resulting in a more marked decrease of the skin severity score than that achieved with betamethasone alone. This synergistic effect was also superior to treatment with tetracycline alone, which would have been attributable to not only the reduced lesional *S. aureus* count, but also the synergistic immunosuppressive effects of the two drugs. The standard treatment for AD is mainly topical steroids, and topical antibiotics are generally not actively applied for the disease unless impetigo is present in the skin lesions. However, the present observations suggest that in order to fully heal AD, both an antibacterial action against *S. aureus* on the lesional skin and inhibition of excessive Th1/Th2 cell development are necessary. Therefore, irrespective of the presence of impetigo, if skin lesions are colonized by *S. aureus* and are sensitive to tetracycline, aggressive use of the latter in topical form along with betamethasone ointment would be a more effective treatment strategy for AD.

## Conclusion

Our results have demonstrated that combined topical application of betamethasone and tetracycline can synergistically inhibit the development of AD-like skin lesions and the Th1/Th2 immune response in NC/Nga mice through both immunomodulative and bactericidal actions. This dual therapy appears to have potential as a new strategy for AD patients with superficial *S. aureus* colonization.

## Data Availability

The original contributions presented in the study are included in the article/supplementary material, further inquiries can be directed to the corresponding author.
